# Editorial: Scaling the Turbulence Edifice (part 2)

**DOI:** 10.1098/rsta.2021.0102

**Published:** 2022-03-21

**Authors:** Jérémie Bec, Giorgio Krstulovic, Takeshi Matsumoto, Samriddhi Sankar Ray, Dario Vincenzi

**Affiliations:** ^1^ Université Côte d’Azur, INRIA, CNRS, CEMEF, Sophia-Antipolis, France; ^2^ MINES ParisTech, PSL Research University, CNRS, CEMEF, Sophia-Antipolis, France; ^3^ Université Côte d’Azur, Observatoire de la Côte d’Azur, CNRS, Laboratoire Lagrange, Boulevard de l’Observatoire CS 34229, NICE Cedex 4 06304, France; ^4^ Division of Physics and Astronomy, Graduate School of Science, Kyoto University, Kitashirakawa Oiwaketyo Sakyoku, Kyoto 606-8502, Japan; ^5^ International Centre for Theoretical Sciences, Tata Institute of Fundamental Research, Bangalore 560089, India; ^6^ Université Côte d’Azur, CNRS, LJAD, Nice, France

**Keywords:** turbulence, inviscid limit, mixing, closure models

## Abstract

This is the second part of a two-part special issue of the Philosophical Transactions of the Royal Society A, which recognizes, and hopefully encourages, the growing convergence of interests amongst mathematicians and physicists to scale the turbulence edifice. This convergence is explained in more detail in the editorial which accompanies the first part (Bec *et al*. 2022 *Phil. Trans. R. Soc. A* **380**, 20210101. (doi:10.1098/rsta.2021.0101)) and includes a tribute to our friend, collaborator and mentor Uriel Frisch, to whom these special issues are dedicated. Uriel, the principal architect of the Nice School of Turbulence, remains the finest example of this synthesis of mathematics and physics in tackling the outstanding problem of turbulence.

This article is part of the theme issue ‘Scaling the turbulence edifice (part 2)’.

The collection of papers selected in this volume constitutes the second part of the theme issue of the Philosophical Transactions of the Royal Society A *Scaling the Turbulence Edifice*. As in part 1, the contributing papers gathered here come from the physics and mathematics communities and have a common interest in the big problem of turbulence.

One of the difficulties encountered when providing a statistical description of turbulence is the emergence of an infinite hierarchy of equations for the evolution of velocity moments. Owing to the intrinsic nonlinear character of turbulence, and hence the lack of a small parameter, finding closures to such hierarchies has challenged, for decades, not only theoretical physicists and mathematicians but also engineers and applied physicists. One of the goals has been to understand and model small-scale turbulent fluctuations and to comprehend their effect on the large scales of the flow. In the first paper of part 2, Kurien & Pal present a review on a two-point closure known as the Local Wavenumber model, which was introduced more than 30 years ago. Then, Flandoli, Galeati and Luo give the proof of the existence of an *eddy-diffusivity* constant in an idealized model of turbulent convection in the presence of boundaries. The following work by Biferale & Alexakis describes the statistical behaviour of the λ-Navier–Stokes model, in which the type of helical interaction can be tuned by using a control parameter.

As is well known, the limit of vanishing viscosity of the Navier–Stokes equations presents deep mathematical difficulties, as their limiting solutions are not necessarily solutions of the Euler equation in the strong sense. Lopes Filho & Nussenzveig report a mathematical study on weak solutions of the forced two-dimensional Euler equations, for which they prove an energy balance relationship. Then, Brenier & Moyano study the initial value problem in a relaxed Euler equation, the multi-stream pressure-less gravitational Euler–Poisson equation. Continuing with pressure-less fluid systems, Khanin & Li report a study on the randomly forced Burgers equation and its connection with the end-point distribution for directed polymers. Then Cartes, Tirapegui, Pandit and Brachet study the dynamics of the one-dimensional Galerkin-truncated Burgers equation and show that different scaling laws for the correlation time function emerge depending on the ultraviolet cut-off.

Part 2 of the theme issue *Scaling the Turbulence Edifice* also covers topics that go beyond hydrodynamic turbulence described by the Navier–Stokes equations and related models. In the review article presented by Pouquet & Yoi, the authors describe the role of helicity in turbulent magnetohydrodynamic flows. They discuss its effect on the physics of magnetic reconnections and how to construct models of turbulence for helical flows. Then, L'vov, Lvov, Nazarenko and Pomyalov present a new theory to describe the anisotropy of a turbulent counterflow of superfluid ${\;}^{4}\mathrm{He}$, which is described by a set of coupled Navier–Stokes equations. Three papers are devoted to describing the physics of systems where a turbulent flow transports some scalar field. Bhattacharjee analyses stably stratified fluids and shows that the so-called Bolgiano–Obukhov scaling is observed, but presents a strong anisotropy. Then, Boffetta & Musacchio study Rayleigh–Taylor mixing in a confined domain and show how mixing is enhanced in bulk flow and porous media. Mazzino & Rosti present a work related to transported scalar fields. They numerically investigate the evolution of a puff and validate a recent phenomenological model which describes its evolution. They focus on a new regime where turbulent fluctuations are dominated by buoyancy. Finally, the last paper of part 2 is devoted to Lagrangian dynamics. Bhatnagar, Pandey, Perlekar and Mitra consider the formation of caustics in heavy inertial particles advected by a turbulent flow. In particular, by performing extensive numerical simulations of two- and three-dimensional turbulent flows, they characterize the rate of caustic formation as a function of the Stokes number.

The work compiled in this theme issue covers a broad range of turbulent research. We expect that this collection will trigger a renewed dialogue between the communities of physics and mathematics.

## Acknowledgements

We are indebted to the authors whose work adorns this issue. We are most grateful to them for their enthusiasm for this project, when we first reached out to them, and then for accepting and honouring the timeline that we imposed. Thank you.

We also thank our friend and colleague John D. Gibbon of the Imperial College London who suggested to us the title of this issue and our many colleagues to whom we had reached out to referee and shape the final form of the articles. We are indebted to our colleague Siddhartha Mukherje, at the International Centre for Theoretical Sciences, Tata Institute of Fundamental Research (ICTS-TIFR), Bangalore, for making the wonderful covers, which are visualizations of a dissipative Euler flow, that embellish both these issues.

Finally, all of this would not have been possible without the support of the Royal Society Publishing and in particular the Editorial Office of the Philosophical Transactions of the Royal Society A. We would like to place on record the help, patience, advice and leadership, at every step of the way, of the Commissioning Editor, Alice Power. Alice has been most kind and generous with her time and we are truly grateful to her. We also thank Helen Eaton and Jackie Knapp who stepped in every time we needed help.

## Data accessibility

This article does not contain any additional data.

